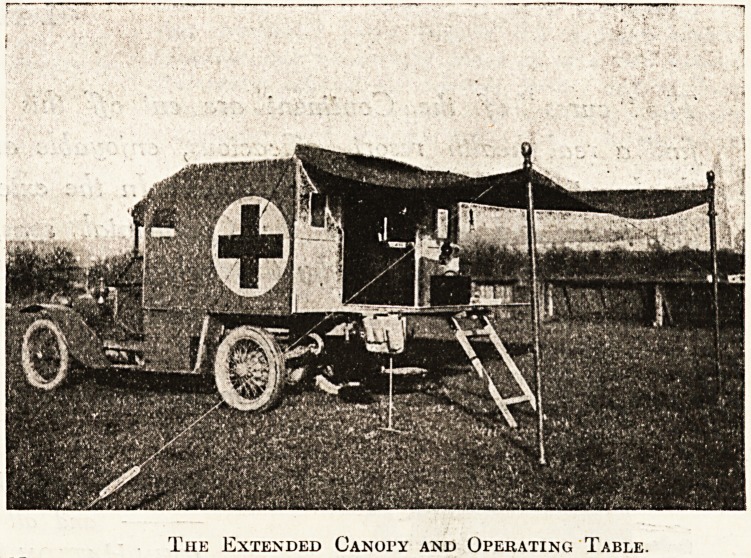# Institutional Needs

**Published:** 1915-04-17

**Authors:** 


### INSTITUTIONAL NEEDS.

### ECONOMY AND SAFETY BY KITCHEN MACHINERY.

It is no exaggeration to say that a potato-peeling machine of one kind or another is now used in every up-to-date establishment where a fairly large quantity of potatoes are used per day. Never was this more the case than at present, when economy in the kitchen, always advisable, has now become a necessity. Not only do these machines save material in the form of potatoes, but time and labour, two considerations which no institution relying upon voluntary effort for its support can nowadays afford to ignore. Messrs. Mabbott and Company's potato-peeling and bread-cutting machines are obtainable in all sizes and prices ranging from £3 to £36, and any quantity of potatoes, from 6 lb. to 40 lb. per minute, can be properly peeled in them. The special advantages of their design include the following: They can be supplied either for hand power or complete with electric motor. It may be pointed out that the only part of the work that has to be done by hand is the putting of the potatoes into the machine. The rest of the work is all done automatically, and includes the delivery of the potatoes and the removal of the waste and water. The largest machine only occupies a space of 2 feet 6 inches square. The smallest machine, the No. 3 "Rapid," which is much used, will peel 6 lb. per minute, and can stand on a table occupying a space of about 18 inches square. The firm have supplied these machines in very large quantities both to the Army and Navy and to public institutions throughout the country.

In Messrs. Mabbott's bread-cutting machines the bread is moved up to the knife automatically, thus avoiding a possible chance of accident. The value of this device on humane and legal grounds is one which needs no special emphasis to institutional managers. Many people know from experience what a risk there is with the old type of machine where the bread is pushed up by the hand. Indeed, to make the machine fool-proof, not only is the bread moved up automatically, but the blade itself is encased in a shield so that the operator cannot come into contact with it. The machine will cut any thickness of slice from an eighth of an inch to one inch thick. A small platform is arranged at one end of the machine, so that as the slices are cut they are stacked and not allowed to fall on to the table. Such a fall frequently results in broken slices of bread, and therefore waste, especially in the case of a thin slice, which, if allowed to fall on to the table as by many machines, is almost invariably broken. The blade can be easily sharpened without removing it from the machine. The whole construction of the machine is simple, and the firm's claim that their machine is a practical economy is borne out by the obvious fact that users are able to get many more slices per loaf out of all bread which is sliced by it.

### THE MOVING OF HELPLESS CASES.

That the patent invalid-lifters made by Mr. Skeffington, of 49 Ulundi Road, Blackheath, S.E., are becoming more and more used in hospitals and nursing homes is a circumstance which is not a matter for surprise when the advantages of these lifters in nursing helpless cases are realised. For chronic cases in private houses an invalid-lifter may be not only a great comfort, but its use will often obviate the necessity for a second nurse, or even allow the friends to do without extraneous help altogether. The "Skeffington" apparatus is easily adjusted to a bedstead, and is made in three types; the methods of employment are easily understood if the excellent diagrams in Mr. Skeffington's catalogue are consulted. Among the objects which this apparatus aims at effecting are the prevention of a weak patient from slipping down in the bed; the raising of a patient into a sitting posture, and maintaining him there without exertion on his part; the lifting a patient bodily, hammockwise, above the bed in order to change sheets or to give a bed-pan. If proof were needed to show the practicability of these appliances and the good opinion of them held by hospital authorities, one might instance the experience of the Ilford Emergency Hospital, in which Mr. Skeffington's sacrum lifter was employed for cases injured in the recent railway accident. His patent invalid-lifters, it is understood, have also been supplied to the War Office, and are being used at the Red Cross Hospital, Netley, and at the Queen of the Belgian's Hospital, Wimereux. Full particulars of these appliances, which can also be had on hire, will be forwarded on application to Mr. Arthur Skeffington, 49 Ulundi Road, Blackheath.

### A MOTOR FIELD OPERATING VAN.

To the articles contained in this issue should be appended a description of the motor field operating van, which has been built from the designs of Surgeon-Captain Wade, in collaboration with Messrs. Atkinson and Philipson, of Newcastle, the makers. It should be noticed that this van is distinct from the motor operating theatres in the fact that it is suitable for a light chassis, and does not require the long body and heavy chassis which are a necessity for the motor operating theatre. The principle of compactness upon which Mr. Wade and Messrs. Atkinson and Philipson have proceeded enables the surgeon to carry out the operations under an extended canopy at the rear of the van; and it is confidently claimed that this saving of space and weight afford equally good facilities to those of a room inside the van. The canopy is carried on a light iron frame, which slides from under the roof. The interior of the van is lined with aluminium on the sides and asbestos on the front. An anæsthetic cabinet and an instrument cabinet occupy each side, and sterilisers the front. Primus burners are used. There is also a rack for screens, a copper spirit tank, a patent case in which compressed bandages are stored, and a wash-basin is also fitted. The interior of the van is carefully lighted, and the actual operating table, which is covered with aluminium, slides out on a frame, as shown in the illustration, fitted with a tray for surgical instruments and an anæsthetist's bag. The table is lighted by two powerful acetylene lamps. The medical branch of the Scottish Horse, for whom Captain Wade has designed this light field van, should have a very useful addition to its resources, one which can be conveniently and swiftly handled, and which is compact without being cumbersome.

The Extended Canopy and Operating Table.

Closed View of the Motor Operating Van.

### THE "PARAGON" BED TABLE AND "ECLIPSE" READING STAND.

Probably there is no article of ward equipment more useful than an up-to-date bed table, and the demand has led Messrs. W. H. Bailey and Son, 38 Oxford Street, London, W., to make a speciality of the "Paragon" patent bed table, of which numbers are understood to be employed. In the "Paragon" table all the metal parts are of malleable castings and steel tubing, thus breakages are impossible as compared with similar tables on the market made of ordinary cast iron. The tables bring comfort and ease to patients, and nurses are saved many steps and much inconvenience. The feet of the table go underneath the bed out of the way, and the table top does not touch the bed, but passes over it. It can be raised to any height and tilted to any angle or position in an instant, and will remain firm wherever placed. A

collapsible nickelled device for holding books is attached to the moulding on each side of the table, and a telescopic side attachment carrying a small reading slope can be supplied if required. Messrs. Bailey and Son are old-established providers of up-to-date hospital appliances, and their "Paragon" table sustains their reputation.

The "Eclipse" reading stand is another very useful article supplied by Messrs. Bailey which should favourably appeal to hospitals and nursing homes for those soldiers injured in such a way as to be quite unable to sit up, since a special device can be supplied and attached to the "Eclipse" reading slope enabling a person who cannot sit up to enjoy the delights of reading whilst lying flat on the back. The "Eclipse" certainly claims attention, and we recommend those who are interested to communicate with Messrs. Bailey, who will supply illustrated particulars.

### HAIR-SUBSTITUTE MATTRESSES.

Hygienic bedding is a costly matter, and therefore a welcome is afforded to the "Lyxhayr" mattress, manufactured by G. Watson, City Bedding Works, Bissell Street, Birmingham, which is a hair substitute of vegetable origin. Superior to the cheaper hairs, resilient, and insect proof, the makers point out that "Lyxhayr" is the only substance which can be subjected without injury to the high temperatures necessary to destroy infectious germs. To show the value of this hair substitute, the firm offers to send to any institution a 3-ft. sample "Lyxhayr" mattress on approval for one month's trial. Its qualities are remarkable, and should commend themselves to those now actively engaged in equipping adapted premises or emergency institutions who desire the best and cheapest substitute for hair mattresses of the ordinary kind.

### THE LAVEAU BED TABLE.

The difficulties involved in attending to bedridden patients, especially in emergency hospitals where questions of space and the adaptation of rooms not designed for nursing patients are insistent, can be mitigated by the use of such a compact and ingenious bed table as that made in the English Lake District by the Laveau Bed Table Company, of Hawkshead, Ambleside. It has many uses, and when not employed can be folded up, thus occupying the least possible space. The Laveau bed table and complete outfit consists of the table, and a basin, soap-dish, and two tumblers in British enamelled ware, and is sold at the inclusive price of 30s. or 32s. 6d., according to the wood from which it is made. The utensils fit securely into holes in the table so that it can be conveniently carried, and a tray (which can be tilted to form a reading or writing desk) is made to fit on the wash-table, so that breakfast may be served on it after the washing appliances have been removed. It is understood that many of these Laveau bed tables have been sent to war hospitals in France, and there are few institutions which would not be the better for possessing one. The table, complete with its accessories, will be sent direct from the makers, no charge being made for packing or carriage. It is a highly ingenious design.

### UP-TO-DATE LAUNDRY MACHINERY.

The Cherry Tree Machine Company, Ltd., laundry engineers, Blackburn, are supplying the new laundry machinery, including their latest type metal rotary washing-machines with self-oiling bearings, also self-balancing type hydro extractor with ball-bearing footstep and self-oiling bearings, to the Epileptic Colony, Langho, near Blackburn, for the Chorlton and Manchester Joint Asylum Committee.

### Bath Water Treatment and the War.

Bath has been growing in popularity, and the largely increased number of visitors this spring is undoubtedly due to a considerable extent to the war; for many people who have been accustomed to go to the Riviera, and others who prefer the safety of an inland town, have come to Bath, and many visitors from the East and South Coasts are staying there. Early in the war the War Office and Admiralty were offered free treatment for all wounded or invalided officers and men from the Front who might be benefited by the Bath waters. This offer was accepted, and cases have been sent down. Officers who are sent to Bath for treatment either stay at hotels or in apartments, arranging their accommodation themselves, or with friends resident in Bath, and in some cases private hospitality has been given through the Red Cross Society.

It was found desirable to open a convalescent home for wounded and invalided officers coming to Bath for mineral-water treatment. This has been organised by Lady de Blaquiere at 27 and 28 Marlborough Buildings. The house is now open, and is extremely comfortable. Cases for this convalescent home are sent through the Headquarters of the Red Cross Society, and application is made to the Countess of Dudley. The Royal Mineral Water Hospital is also being used for wounded and invalided N.C.O.s and men who are sent to Bath for the treatment of the waters. This hospital is now practically set apart for military cases, and application for admission should be made direct to the Registrar. In all cases application for treatment should come from a medical officer of the Army or Navy, otherwise the Bath authorities have no guarantee that cases are actually from the Front. It is understood to simplify matters if all applications for officers' treatment are made direct to Mr. John Hatton, Director of the Baths, Bath, and all those for N.C.O.s and men to the Registrar, Royal Mineral Water Hospital, Bath.

## Figures and Tables

**Figure f1:**
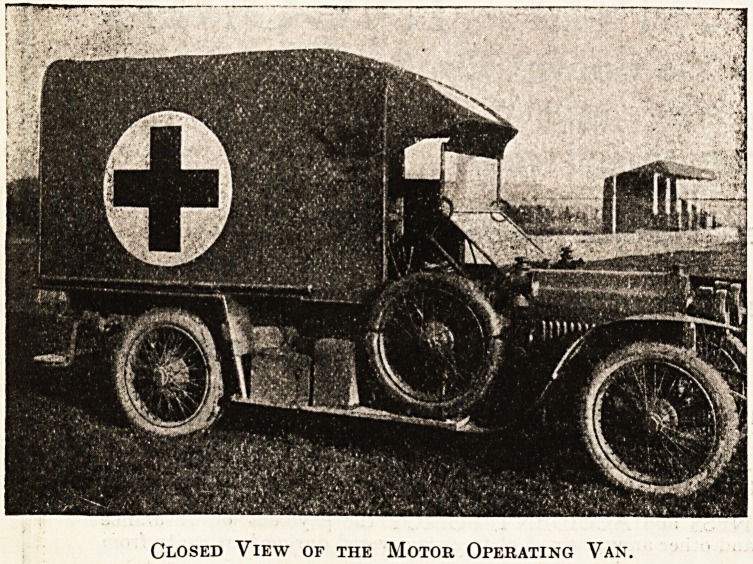


**Figure f2:**